# Biology of Oestrogen-Receptor Positive Primary Breast Cancer in Older Women with Utilisation of Core Needle Biopsy Samples and Correlation with Clinical Outcome

**DOI:** 10.3390/cancers12082067

**Published:** 2020-07-27

**Authors:** Ruth M. Parks, Mohammad Albanghali, Binafsha M. Syed, Andrew R. Green, Ian O. Ellis, Kwok-Leung Cheung

**Affiliations:** 1 Nottingham Breast Cancer Research Centre, University of Nottingham, Nottingham NG7 2RD, UK; ruth.parks@nottingham.ac.uk (R.M.P.); mohammad.aref@bu.edu.sa (M.A.); binafsha.syed@lumhs.edu.pk (B.M.S.); andrew.green@nottingham.ac.uk (A.R.G.); ian.ellis@nottingham.ac.uk (I.O.E.); 2 Public Health Department, Faculty of Applied Medical Sciences, Albaha University, Al Bahah 65779-7738, Saudi Arabia; 3 Medical Research Centre, Liaquat University of Medical & Health Sciences, Jamshoro 76090, Pakistan

**Keywords:** core needle biopsy, tissue microarray, primary breast cancer, older women

## Abstract

The majority of biological profiling studies use surgical excision (SE) samples, excluding patients receiving nonsurgical and neoadjuvant therapy. We propose using core needle biopsy (CNB) for biological profiling in older women. Over 37 years (1973–2010), 1 758 older (≥70 years) women with operable primary breast cancer attended a dedicated clinic. Of these, 693 had sufficient quality CNB to construct tissue microarray (TMA). The pattern of biomarkers was analysed in oestrogen receptor (ER)-positive cases, using immunohistochemistry and partitional clustering analysis. The biomarkers measured were: progesterone receptor (PgR), Ki67, Epidermal Growth Factor Receptor (EGFR), Human Epidermal Growth Factor Receptor (HER)-2, HER3, HER4, p53, cytokeratins CK5/6 and CK7/8, Mucin (MUC)1, liver kinase B1 (LKB1), Breast Cancer Associated gene (BRCA) 1, B-Cell Lymphoma (BCL)-2, phosphate and tensin homolog (PTEN), vascular endothelial growth factor (VEGF), and Amplified in breast cancer 1 (AIB1). CNB TMA construction was possible in 536 ER-positive cases. Multivariate analysis showed progesterone receptor (PgR) (*p* = 0.015), Ki67 (*p* = 0.001), and mucin (MUC)1 (*p* = 0.033) as independent predictors for breast-cancer-specific survival (BCSS). Cluster analysis revealed three biological clusters, which were consistent with luminal A, luminal B, and low-ER luminal. The low-ER luminal cluster had lower BCSS compared to luminal A and B. The presence of the low-ER luminal cluster unique to older women, identified in a previous study in SE TMAs in the same cohort, is confirmed. This present study is novel in its use of core needle biopsy tissue microarrays to profile the biology of breast cancer in older women.

## 1. Introduction

The biology of breast cancer in older women differs compared to younger equivalents; older women are more likely to have cancers that are oestrogen receptor (ER)-positive [1,2,3] and demonstrate less aggressive features [4,5,6,7]. Therefore, although surgery remains the recommended initial treatment for primary breast cancer regardless of age, older women may access endocrine treatment options. A UK national audit found that 40% of breast cancer patients aged >70 years and 55% aged >80 years were treated by primary endocrine therapy (PET) [8].

The majority of studies to date that have profiled the biology of breast cancer have used surgical excision (SE) specimens to do so. This creates potential bias in having not included the large group of patients who have PET and, furthermore, cannot provide any insight when considering neoadjuvant therapy. This present study has, therefore, chosen to focus on ER-positive breast cancer. As an alternative to SE profiling, core needle biopsy (CNB), which is usually obtained at diagnosis in breast cancer patients, should be considered for the study of tumour biology. CNB samples can be obtained from all older patients diagnosed with breast cancer irrespective of primary treatment. Furthermore, preservation of CNB tissue leads to faster penetration of the tissue by a fixative agent when compared to SE, resulting in less chance of enzyme degradation and, thus, better preservation of biological features [9,10].

The technique of tissue microarray (TMA) for use in breast cancer was first reported by Kononen et al. [11] in 1998. The construction of TMAs involves embedding multiple fragments of tumour tissue in a single paraffin block for the purpose of high-throughput analysis. In addition to maximising tissue resources, TMA has the advantage of facilitating evaluation of tissue-based assays in an efficient, cost-effective, and uniform manner [12]. It has, therefore, become standard technique to examine tissue biology in detail but primarily in SE specimens, which are more readily available and technically easier to construct from.

There are many challenges faced when manipulating TMA techniques to utilise CNB for profiling tumour biology, including complex construction, given the small diameter of the biopsy and erosion of biopsy after sectioning for initial diagnosis. After a comprehensive systematic literature review on the subject [13], a successful optimal technique to construct TMAs from CNB samples has been developed by the Nottingham group [14]; thus, giving the unique opportunity to utilise CNB for profiling tumour biology. Representative haematoxylin and eosin (H&E) stains from the constructed CNB TMAs are given in Figure S1.

The overarching goal of this study was to investigate—using CNB TMAs—the patterns of biomarker expression in ER-positive primary operable breast cancer in older women and the relationship with clinical outcome. Breast-cancer-specific survival (BCSS) was the measured clinical outcome as it served as a ‘surrogate’ for tumour biology, not influenced by competing causes of death due to comorbidity, which could be significant in the older population.

The study goal was achieved through the following steps, based on examining the CNB TMA cohort:

1. Consideration of standard clinicopathological features in the cohort.
2. Measurement of a panel of biomarkers and correlation of findings with BCSS.
3. Performing cluster analysis of the measured biomarkers and correlation of findings with BCSS.

## 2. Results

### 2.1. Clinicopathological Features

A summary of characteristics for ER-positive samples (*N* = 536) is given in Table 1.

### 2.2. Panel of Biomarkers

Table 2 demonstrates the expression of biomarkers in this series. Representative marker stains are demonstrated in Figure S2.

### 2.3. Panel of Biomarkers—Correlation with BCSS

Univariate analysis found that positive expression of progesterone receptor (PgR) (*p* = 0.006), VEGF (*p* = 0.009), and BCL-2 (*p* = 0.021) were associated with longer BCSS, whereas positive expression of Ki67 (*p* ≤ 0.001), MUC1 (*p* = 0.006) and p53 (*p* ≤ 0.001) were associated with shorter BCSS. Results of univariate analysis are depicted in Figures S3–S8.

The Cox regression model for multivariate analysis of biomarkers expression indicated that PgR, Ki67, and MUC1 were independent predictors for BCSS in older women with ER-positive early breast cancer (Table 3).

### 2.4. Cluster Analysis

**Figure 1:**
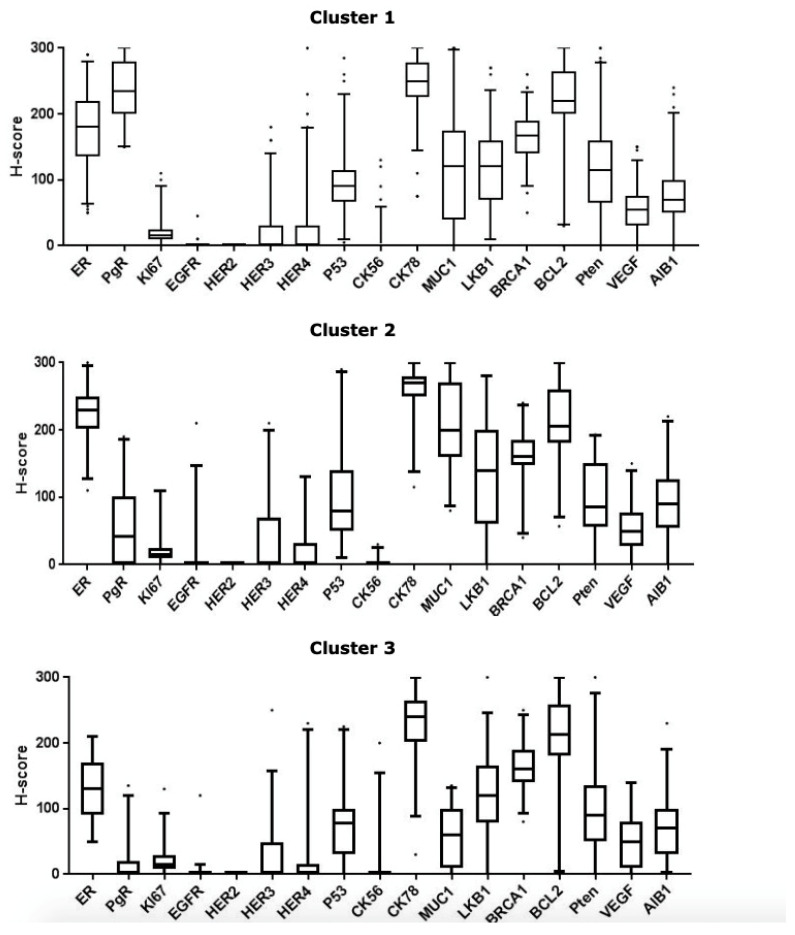
Boxplots used to define patterns of ER-positive early operable primary breast cancer in older women, demonstrating 3 clusters including 17 biomarkers. The middle line within each box indicates median expression whilst outliers are indicated using dots.

Including the H-scores of the 17 biomarkers as above, cluster indices suggested three clusters (Figure 1) showing a moderate agreement between K-means and partitioning around medoids (PAM) methods (Kappa coefficient = 0.51).

Cluster 1 had a high expression of ER, PgR, CK7/8, BRCA1, and BCL-2 and low expression of Ki67, EGFR, HER2, HER3, HER4, p53, CK5/6, MUC1, LKB1, PTEN, VEGF, and AIB1. In comparison, Cluster 2 had a lower expression of PgR and higher expression of MUC1 and ER compared to Cluster 1. The third cluster had higher expression of CK7/8, BRCA1, and BCL-2 and a lower expression of ER compared to Clusters 1 and 2.

The biomarker array of Cluster 1 was consistent with luminal A-type tumours, and Cluster 2 was consistent with luminal B. The third cluster was consistent with the low-ER luminal cluster, which has been previously demonstrated in surgical excision samples in older women [7]. The clusters will subsequently be referred to as luminal A, luminal B, and low-ER luminal.

### 2.5. Cluster Analysis—Correlation with BCSS

**Figure 2:**
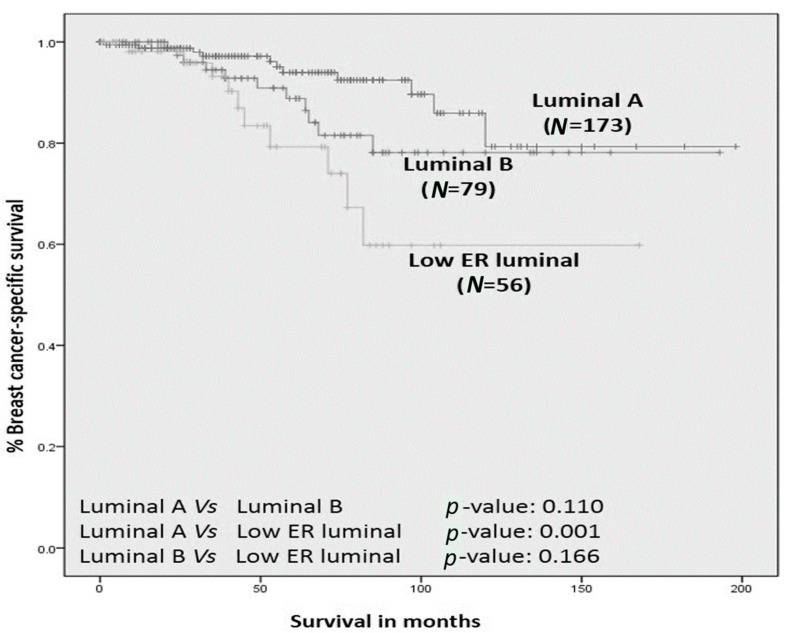
Older women with oestrogen receptor positive primary breast cancer: breast-cancer-specific survival of biological clusters.

The 5-year BCSS for all patients in Luminal A cluster, Luminal B cluster, and low-ER luminal cluster, respectively, was 93%, 87%, and 73% (Figure 2).

Luminal A cluster had significantly better BCSS compared to low-ER luminal cluster (*p* = 0.001). There were no other significant differences in BCSS between other cluster comparisons.

**Figure 3:**
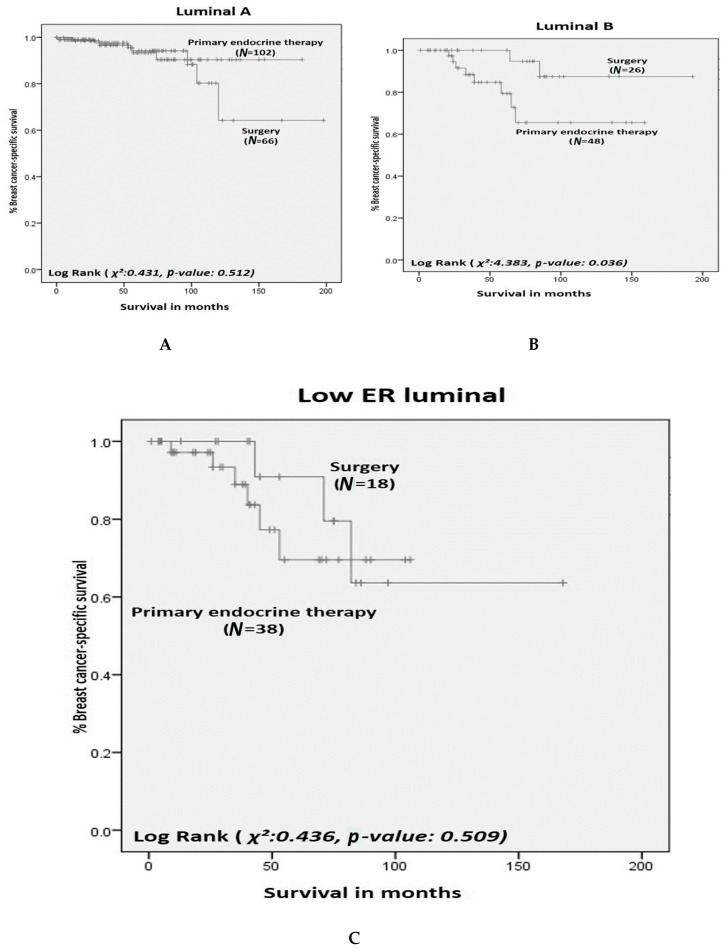
Older women with oestrogen receptor positive primary breast cancer: breast-cancer-specific survival of biological clusters (**A**) luminal A, (**B**) luminal B, and (**C**) Low-ER luminal according to surgery versus primary endocrine therapy.

Within the clusters, patients treated by surgery had a 5-year BCSS of 88% (luminal A), 95% (luminal B), and 80% (low-ER luminal), respectively, and, for PET patients, 5-year BCSS was 90%, 73%, and 70%. There was no difference in BCSS between patients treated by PET or surgery for patients in luminal A or low-ER luminal clusters (Figure 3).

In contrast for patients in luminal B cluster, BCSS was better in patients treated with surgery compared to PET (*p* = 0.036).

## 3. Discussion

### 3.1. Summary of the Findings

In this cohort of 538 women aged ≥70 years with ER-positive primary operable breast cancer, it was possible to construct TMAs from CNBs (taken at diagnosis). From a panel of 17 biomarkers PgR, Ki67, and MUC1 have been shown to have independent prognostic significance. A novel biological cluster (‘low-ER luminal’), previously shown in the older population using SE TMAs [7], was confirmed.

### 3.2. CNB TMA

Using a unique technique developed by the Nottingham group, this present study has confirmed that it is possible to construct TMAs from CNB samples, rather than using the traditional approach of SE samples. There are few existing studies in the literature that have utilised this technique, mainly in the context of prostate cancer [13] and with far fewer biomarkers (maximum of three per study) than tested in this present study. This is a major breakthrough in the field of breast cancer and indicates that the biology of breast cancer can be fully characterised regardless of primary treatment. This present study is the first study of its kind to examine a large panel of 17 biomarkers in the context of breast cancer in older women.

### 3.3. Clinicopathological Features

The age range of patients in the cohort was 70–99 with a median of 79 years. Of these, more patients were treated with PET rather than surgery. This is a higher rate of PET than previously documented in the literature, which estimated around 40% uptake of PET in older women [8,15]. This could partly be historical, as the study covered over two decades. Breast-cancer-specific survival for the whole cohort was in keeping with national data [16]. As the overarching project goal was to characterise breast cancer in older women regardless of primary treatment, the authors feel that this current cohort is representative of this population.

### 3.4. Measurement of Panel of Biomarkers and Correlation with Clinical Outcome

The biological markers PgR, BCL-2, Ki67, MUC1, p53, and VEGF were found to be predictive of BCSS in univariate analysis, while pooled analysis of these markers retained the independent prognostic significant of PgR, Ki67, and MUC1 on a multivariate model.

These findings mainly correlate with those of other studies examining expression of biomarkers in all age groups. The importance of PgR expression has been reported to have strong prognostic significance in studies involving younger breast cancer patients and is linked to risk of relapse and poor survival [17,18]. Ki67 has been shown to have significant value in predicting BCSS [19,20]. MUC1 overexpression is associated with hormonal-therapy resistance in ER-positive breast cancer [21] and worse prognosis [22] in patients with MUC1 positive expression.

There is a lack of consensus concerning the optimal number of cancer cells needed to achieve reliable Ki67 results [23]. The authors appreciate that it may be difficult to account for sample heterogeneity when using small tissue specimens provided by CNB, however by using the TMA method we have a much larger amount of tissue for analysis, so this concern is minimised.

In a previous study conducted by the Nottingham group, measuring biomarkers on SE TMA [7] in the same cohort of patients, findings relating to biomarker expression were the same as this present study, with the exception of VEGF. In the SE TMA study, there was a nonsignificant pattern of poor survival in the VEGF positive group, which is contrary to findings in this present study. In the previous study [7] the expression of all biomarkers was compared between the older women cohort and a comparative younger (<70 years) cohort; differences in expression of biomarkers between the two cohorts was found.

These present findings add to the growing body of evidence that the biology of primary operable breast cancer in older women displays less aggressive biological features when compared to their younger counterparts [7,24,25]. From the overall literature, primary breast cancers in older women are more likely to be ER-positive, express luminal cytokeratins and have lower markers of proliferation [7,24,25]. The literature shows difference between biomarkers expression in the younger population [7,26,27,28]. Given the differential expression of these biomarkers in the older population, and the fact that they retain the same prognostic significance, it is important to personalise treatments in this population based on accurate assessment of the detailed tumour biology.

Although the expression of biomarkers varies with age, the prognostic significance of expression remains. This supports the clinical need for a personalised-treatment approach in older women based on their individual breast cancer biology.

Some tools to help inform the prognosis and response to therapy do exist. The Nottingham Prognostic Index [29] was the first tool of its kind to assess a combination of factors, including tumour grade, size, and nodal status, to inform prognosis following surgery. Assessment that is more comprehensive, for example Adjuvant! Online [30], which uses more clinicopathological features, has been developed, but recruitment of older women in their conception is lacking. Furthermore, the aims of these tools are not focused specifically for the treatment of older women and do not necessarily take into consideration unique tumour material from a patients’ tumour, therefore they do not providing truly individualised therapy. Finally, the majority of these tools assess prognosis after surgery, limiting their use in the older population who receive PET.

### 3.5. Cluster Analysis and Correlation with Clinical Outcome

This study has identified three biological clusters. Two of the clusters are consistent with well-known subtypes; luminal A and B [31]. The low-ER luminal cluster has previously been shown to be a unique cluster to primary breast cancer in the older woman compared to the younger cohort (<70 years), based on our work using SE TMAs, as described above [7]. Cluster analysis in the SE TMAs constructed showed 6 clusters: luminal A, luminal B, low-ER luminal, basal-like, all low expression, and HER-2 positive. As the cohort in this present study is in patients with ER-positive cancers only, we do not expect to see the basal-like, all low expression, or HER-2 positive clusters.

Luminal A patients respond well to both surgery and PET; however, luminal B patients do not respond to PET as well as their counterparts having surgery. Luminal B-like tumours have lower expression of ER and PgR compared to luminal A-like and higher histological grade tumours [32], which may explain this finding.

Although the low-ER luminal cluster has lower BCSS compared to luminal A and B in our study, response to surgery or PET within this cluster is similar. This could be interpreted as both treatments being equally as effective in this cluster, or that both treatments are equally unsatisfactory to treat this group of patients. This is an interesting finding and may suggest that—in addition to conventional features, such as grade, nodal status, and ER status—cytokeratin expression may be significant in the role of response to PET. Current literature has explored the association between cytokeratin and prognosis in breast cancer [33,34] but not specifically as a marker of response to PET. It is recognised however, that in this study Cluster 3 contains the smallest number of patients so results may not be applicable in a larger population. Going forward, this finding is worthy of further validation in a larger cohort.

### 3.6. Strengths of the Study

This is the first study of its kind to use CNB TMA to characterise the biology of breast cancer to this extent. Our study confirms that CNB TMA construction is feasible and practical and allows for a large panel of biomarkers to be assessed. This will allow ongoing research in breast cancer regardless of primary or neoadjuvant treatment.

The series described consists of older women with primary operable breast cancer, consecutively treated at a single centre with long-term follow-up data available [35]. Changes in ethical approval practices over the last four decades means that a database of this kind is unlikely to be reproduced in the future. All samples were processed using the same methodology in a single laboratory to allow for uniform techniques as much as possible.

### 3.7. Weaknesses of the Study

The process of CNB TMA is still in its infancy and requires refining to make it more efficient and reproducible. Due to the smaller amount of tissue available from CNB (compared to SE), it was not possible to construct CNB TMA in some cases. No direct comparison has been made between SE TMA and CNB TMA to check for concordance. It is noted that the confidence intervals from multivariate analysis of biomarker expression and relationship to BCSS are quite wide, which is possibly due to relatively smaller sample size and long-term follow-up. It is hoped that, with ongoing refining of the CNB TMA process, this can be improved.

### 3.8. Future Directions

Future work will compare biomarker data from CNB and SE TMAs from the same patients in the whole series of older women with primary breast cancer, including patients with ER-negative tumours [36]. It would be helpful for the technique to be utilised in tumour samples from other centres. Bioinformatics principles and technologies could also help analyse such a large dataset (biomarkers and clinical outcome data) [37] into a format that could be used clinically, e.g., at diagnosis. This may take the form of a new prognostic model.

Competing causes of death contributing to overall survival in the older population must also be considered. One option to assess this is by comprehensive geriatric assessment (CGA), which is designed to detect unique problems in this age group that do not necessarily affect the younger age group. In the future, information gained from both biological assessment and geriatric assessment could be utilised at diagnosis of breast cancer to help guide an older person to make an informed decision regarding treatment.

## 4. Materials and Methods

### 4.1. Patient Group

Over a 37-year period (1973–2010), 1 758 older (≥70 years) women with early operable (<5 cm, T0-2, N0-1, M0) primary breast cancer were managed in a dedicated clinic in Nottingham. This patient cohort has been extensively described [15,35]. To the best of the authors’ knowledge, this cohort is the largest, consecutive series of its kind in the current literature, available for the type of biological analysis described below.

It was possible to obtain diagnostic CNB blocks from 1 221 of the patients in this cohort. Of these, 693 cases had blocks with enough tumour tissue to construct CNB TMAs. From this group, 518 had tumours that were ER-positive, as defined as H-score ≥ 50 [38] on diagnostic CNB (as reported by the histopathologist). A further 18 patients did not have ER status from diagnostic CNB but had tumours that were ER-positive, using the same definition, measured on CNB TMAs, thus bringing the total sample size to 536 for this study. The study was approved by the Nottingham Research and Development committee. The title of the application was ‘Development of a molecular genetic classification of breast cancer’, project registration number: ‘03HI01‘; ethics committee number: C1080301.

### 4.2. Construction of TMA

TMAs were constructed using a novel technique developed, as described [13,14]. Briefly, based on tumour tissue availability, H&E slides and paraffin CNB blocks were reviewed and, multiple areas of 4mm of tumour were marked from each case, retrieved with skin biopsy punch and implanted in Agarose-paraffin TMA blocks.

### 4.3. Clinicopathological Features

Clinical information was available from diagnosis of breast cancer until death or last documented follow-up, as described previously [15,35]. These patients were diagnosed and treated during the period 1984–2010, following the same treatment guidelines at any given time point as previously reported [15]. Briefly, long periods of sample collection treatment protocols were subject to change over time. Measurement of ER status first became part of standard practice at the beginning of the 1990s. All patients with ER-positive disease were offered surgery or PET. Patients with ER-negative tumours were offered surgery or primary radiotherapy. Axillary surgery was only considered when there were clinically palpable lymph nodes. From the early 2000s, the concept of the multidisciplinary team was introduced, and all patients were discussed and seen in a dedicated clinic. The concept of axillary node sampling and later sentinel lymph node biopsy was introduced.

As this work is based on a historical dataset, data on each characteristic may not be available for each patient in the series. Where data is missing, this is classified as ‘unknown’. As ER was introduced into standard practice before PgR and HER2, only ER data is retrieved and reported in this retrospective dataset.

### 4.4. Measurement of Panel of Biomarkers

Immunohistochemical (IHC) staining of 17 biomarkers was performed using StreptAvidin Biotin Complex and EnVision methods (DakoCytomation) [27]. The biomarkers measured were: ER, progesterone receptor (PgR), Ki67, Epidermal Growth Factor Receptor (EGFR), Human Epidermal Growth Factor Receptor (HER)-2, HER3, HER4, p53, cytokeratins CK5/6 and CK7/8, Mucin (MUC)1, liver kinase B1 (LKB1), Breast Cancer Associated gene (BRCA)-1, B-Cell Lymphoma (BCL)-2, phosphate and tensin homolog (PTEN), vascular endothelial growth factor (VEGF), and Amplified in breast cancer 1 (AIB1).

The expression of biomarkers (with the exception of HER2) was assessed using the H-score scoring system (range 0–300) [38]. The Herceptest scoring system [39] was used to score HER2, which involved scoring the staining of the membrane (0–3).

Within a TMA specimen, not all of the samples were equally robust to allow IHC staining for each individual sample. Therefore, only the results of successful staining will be presented.

### 4.5. Cluster Analysis

H-scores of biomarkers from CNB TMAs were utilised to perform cluster analysis. Reported algorithms using unsupervised K-means and Partitioning around Medoids (PAM) were used, as outlined by Soria et al. [40]. This cluster analysis study is a further extension of our previously reported data. The selection of the panel of biomarkers was based on the study that characterised younger patients [27], derived from the Nottingham Tenovus series (2005) and comparison of older and younger series from our group (2013) [7]. The biomarkers showing significant biological relationship in partitioning cluster analysis have been included in this study.

### 4.6. Correlation with BCSS

Breast-cancer-specific survival (BCSS) was calculated from diagnosis to death from breast cancer.

### 4.7. Statistical Analysis

The statistical package SPSS was used for data collection and analysis. The Cox regression model was used to quantify the effect of a biological variable on BCSS. A two-tailed *p*-value of less than 0.05 was determined to be statistically significant. For cluster analysis, the degree of expression of the biomarkers was used as a continuous variable (i.e., 0–300 H-score, where 0 as negative and 300 as 100% positive cells with strong staining). For comparison of the positive or negative expression, the biomarkers were dichotomized by X-tile Bio-Informatics software, which uses the percentage of cells as a continuous variable (i.e., 0–100%) to define positive and negative expressions for that particular database [41].

## 5. Conclusions

This study has shown that CNB comprises an alternate tumour tissue source to SE for biological analysis in older women with primary breast cancer. This is ground-breaking in the field of breast cancer and allows biological analysis of tissue irrespective of primary treatment, which is extremely important in the older population of whom around 40% do not undergo surgery.

This present study adds further evidence that breast cancer in older women exhibits less aggressive biological features compared to their younger counterparts and displays a unique biological cluster (Cluster 3), which has a worse survival outcome compared to patients who identify as luminal A or luminal B.

Further investigation regarding PgR, Ki67, and MUC1 as biomarkers of potential prognostic significance, as well as validation of the findings of a low-ER luminal cluster (Cluster 3), is required. The next steps in the study will be to investigate tumour biology in the ER-negative cohort and compare CNB TMA to SE TMA.

## Figures and Tables

**Table 1:** Patients and tumour characteristics of ER-positive tumours in older women.

Characteristic	Number of Patients	Percentage
*Age (years)*		
70–79	294	55
≥80	242	45
*Year of diagnosis*		
1984–1989	4	1
1990–1994	5	1
1995–1999	174	32
2000–2004	252	47
2005–2010	101	19
*Clinical size of tumour (cm)*		
0.1–2	122	23
2.1–5	162	30
Unknown	252	47
*Grade (from core needle biopsy)*		
1	62	12
2	206	39
3	30	6
Unknown	238	44
*Primary treatment*		
Primary endocrine therapy	333	62
Surgery	189	35
Unknown	14	3
*Stage (Surgery group N = 189)*		
1	93	49
2	63	33
3	14	7
Unknown	19	10

**Table 2:** Percentage expression of biomarkers in the whole population of ER-positive early operable breast cancers in the cohort. Biomarkers measured were: progesterone receptor (PgR), Ki67, Epidermal Growth Factor Receptor (EGFR), Human Epidermal Growth Factor Receptor (HER)-2, HER3, HER4, p53, cytokeratins CK5/6 and CK7/8, Mucin (MUC)1, liver kinase B1 (LKB1), Breast Cancer Associated gene (BRCA)-1, B-Cell Lymphoma (BCL)-2, phosphate and tensin homolog (PTEN), vascular endothelial growth factor (VEGF), and Amplified in breast cancer 1 (AIB1).

Biomarker	Total Samples Tested (*N*)	Positive Expression (%)
PgR	472	78
CK 7/8	499	99.6
BRCA1	400	70
VEGF	468	88
Ki67	471	16
EGFR	479	2
HER2	455	0.2
HER3	498	33
HER4	476	30
P53	477	11
CK5/6	507	12
MUC1	468	23
LKB1	446	31
BCL-2	477	45
PTEN	474	15
AIB1	462	46

**Table 3:** Cox regression model for multivariate analysis of biomarkers for breast-cancer-specific survival in older women with ER-positive early operable primary breast cancer.

Biomarker	*p*-Value	Hazard Ratio	95% CI
PgR	0.015	0.432	0.219–0.850
Ki67	0.001	3.129	1.620–6.042
VEGF	0.209	0.633	0.311–1.291
MUC1	0.033	2.051	1.058–3.976
P53	0.064	2.058	0.960–4.411
BCL-2	0.070	0.539	0.276–1.051

## Supplementary Materials

The following are available online at https://www.mdpi.com/2072-6694/12/8/2067/s1, Figure S1: Representative H&E stains of constructed CNB TMA, Figure S2: Samples from CNB TMAs stained for PgR, Ki67 and p53, Figure S3: Relationship between PgR expression and BCSS (univariate analysis), Figure S4: Relationship between VEGF expression and BCSS (univariate analysis), Figure S5: Relationship between BCL2 expression and BCSS (univariate analysis), Figure S6: Relationship between Ki67 expression and BCSS (univariate analysis), Figure S7: Relationship between MUC1 expression and BCSS (univariate analysis), Figure S8: Relationship between p53 expression and BCSS (univariate analysis).
