# Supplementary material for: Biology of Oestrogen-Receptor Positive Primary Breast Cancer in Older Women with Utilisation of Core Needle Biopsy Samples and Correlation with Clinical Outcome
Source: Cancers (Basel). 2020 Jul 27;12(8):2067. doi: 10.3390/cancers12082067 (PMC7465346; doi:10.3390/cancers12082067)
Supplement: Supplementary file 1 [file cancers-12-02067-s001.pdf]

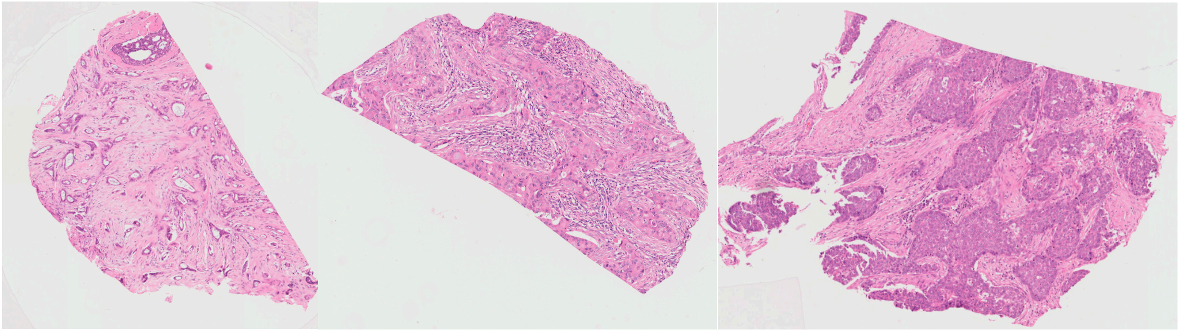

Figure S1. Representative H&E stains of constructed CNB TMA. Representative H&E stains from the constructed CNB TMAs in the described series, are given in Figure A1.

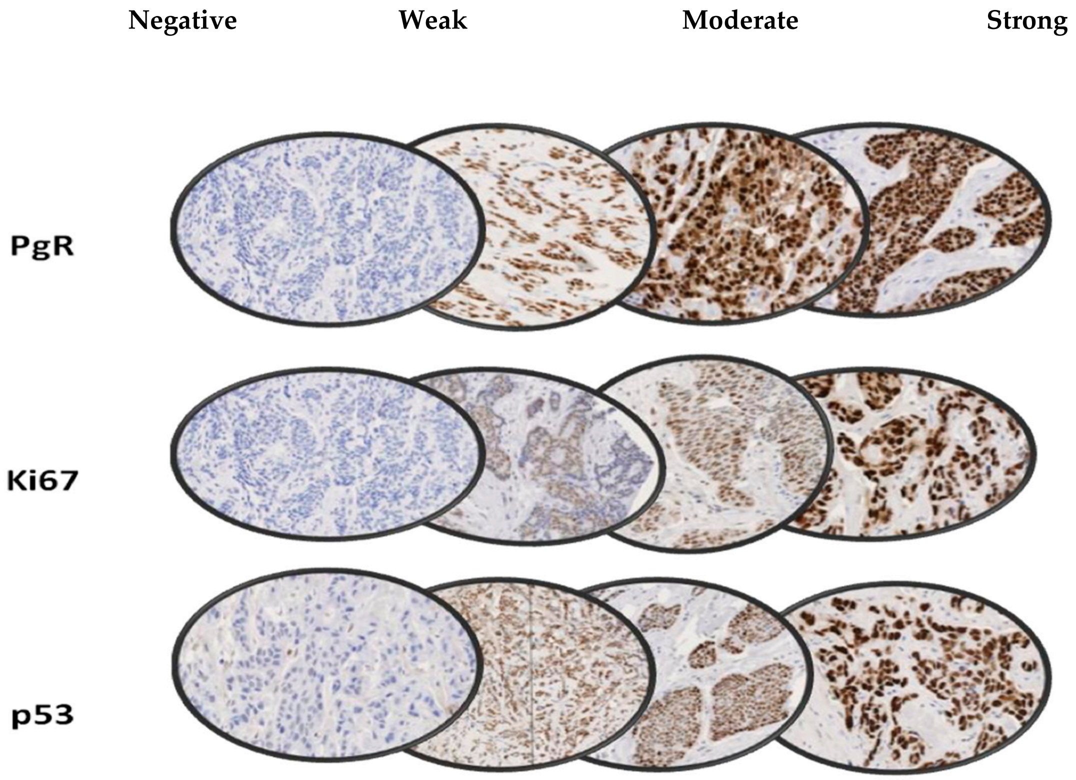

Figure S2. Samples from CNB TMAs stained for PgR, Ki67 and p53. Representative samples of CNB TMAs stained for biomarkers PgR, Ki67 and p53, to demonstrate quality and range of staining, are shown in Figure A2.

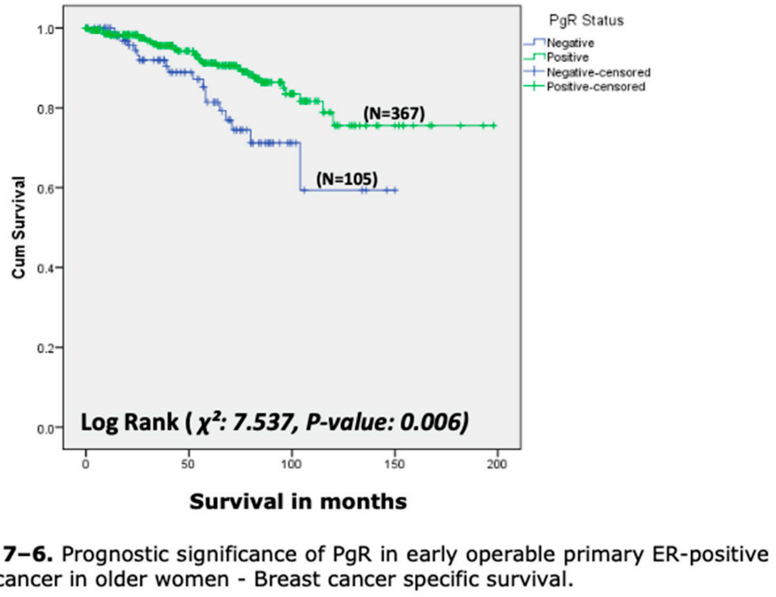

Figure S3. Relationship between PgR expression and BCSS (univariate analysis).

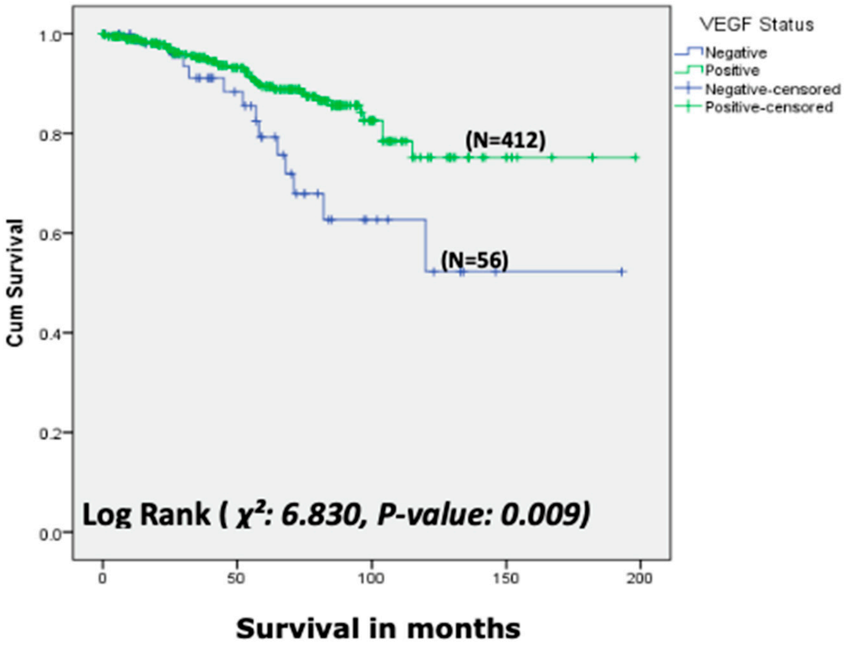

Figure S4. Relationship between VEGF expression and BCSS (univariate analysis).

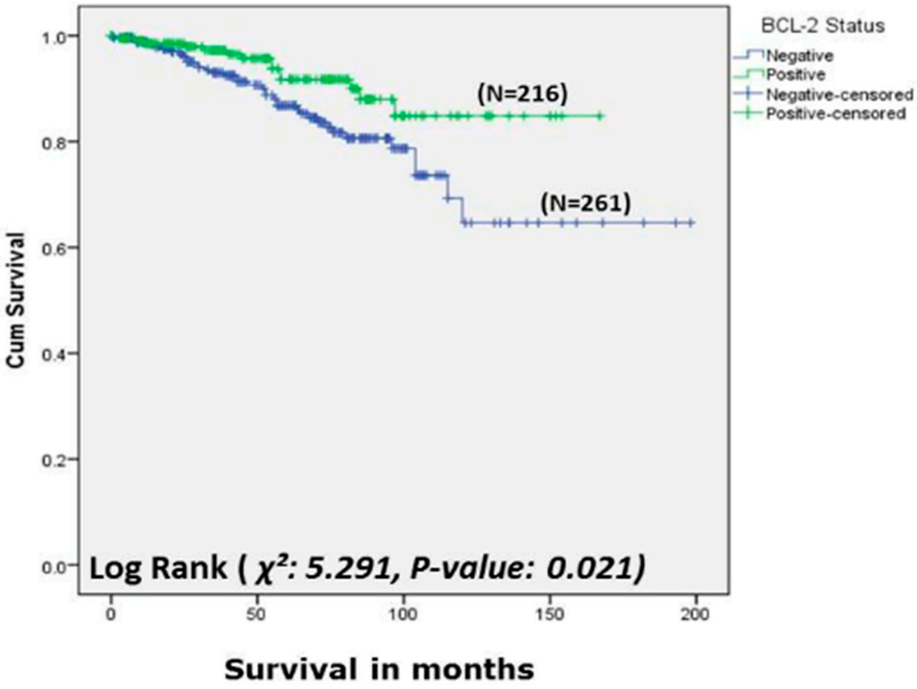

Figure S5. Relationship between BCL2 expression and BCSS (univariate analysis)

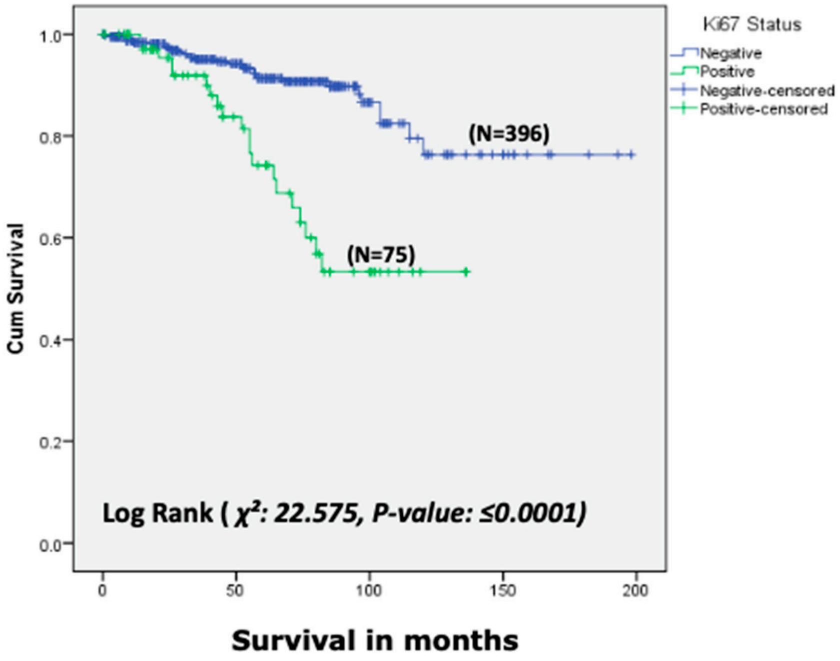

Figure S6. Relationship between Ki67 expression and BCSS (univariate analysis)

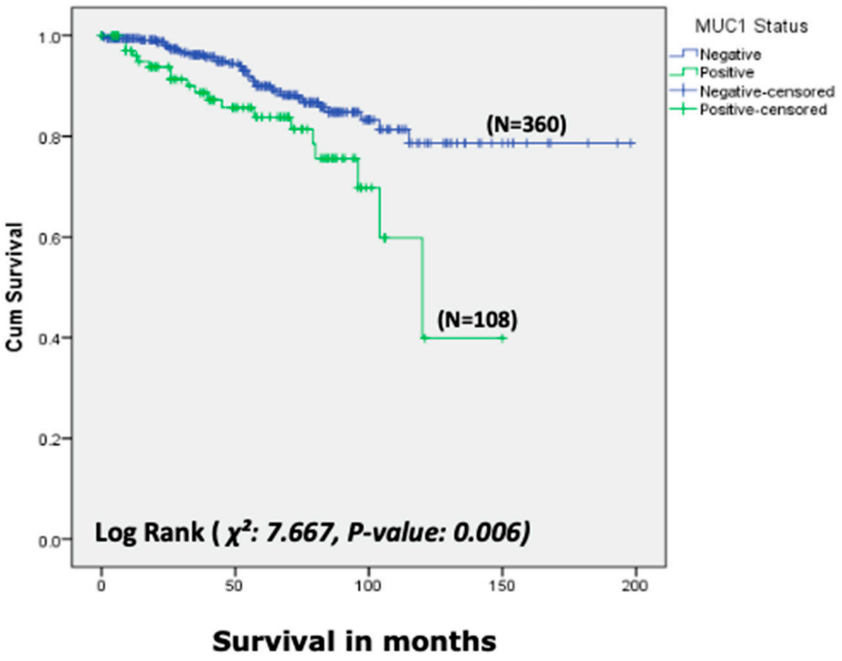

Figure S7. Relationship between MUC1 expression and BCSS (univariate analysis)

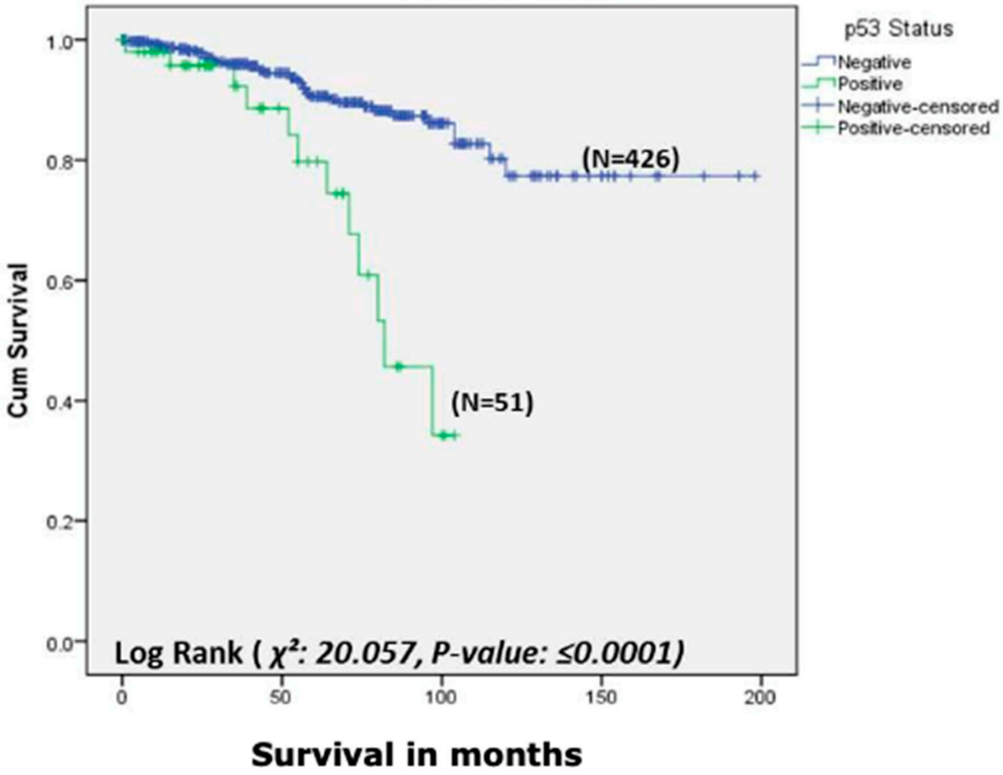

Figure S8. Relationship between p53 expression and BCSS (univariate analysis).
